# Cell survival comparison of proton, helium, and carbon ion interlaced minibeams in water phantom simulations

**DOI:** 10.1002/mp.70516

**Published:** 2026-06-08

**Authors:** Aikaterini Rousseti, Günther Dollinger, Judith Reindl

**Affiliations:** ^1^ Institute for applied physics and measurement technology University of the Bundeswehr Neubiberg Germany; ^2^ Department of physics University of Oslo Oslo Norway

**Keywords:** carbon ion therapy, cell‐survival, dose distribution, helium ion therapy, MONAS, Monte Carlo simulation, particle minibeam radiotherapy, TOPAS

## Abstract

**Background:**

Radiotherapy is a key in cancer treatment, with particle therapy providing better tumor targeting and sparing healthy tissues. Particle Minibeam Radiotherapy (PMBT) integrates the advantages of spatial fractionation into particle radiotherapy by employing submillimeter‐sized beams, thus improving the therapeutic ratio by reducing side effects. Former simulation studies have shown that interlaced proton minibeams from opposing directions in Single Energy Distal‐Edge (1E) mode better protect normal tissue compared to the conventional spread‐out Bragg peak (SOBP) mode.

**Purpose:**

Helium and carbon ion minibeams may be an alternative to enhance the protection of healthy tissue, especially in deeper regions, due to less angular spread. This in silico study evaluates the potential for normal tissue sparing while preserving the same cell survival in the tumor in case of proton, helium and carbon minibeams in 1E mode.

**Methods:**

Simulations were performed using TOPAS (Tool for Particle Simulation) by applying single‐energy interlaced minibeams (beam size σ = 0.2 mm) from two opposing directions in a 250 mm‐thick water phantom, assuming a 50 mm‐thick tumor at the center. For the comparative analysis, cell survival rates were calculated across the whole phantom using the saturation‐corrected Microdosimetric Kinetic Model (MKM‐z*) implemented through MONAS (Microdosimetry‐based modeling for RBE assessment). As a dose constraint, the minimum dose in the tumor was selected to ensure a maximum of 10% cell survival within the tumor. The sparing of healthy tissues was estimated using the Linear Quadratic (LQ) model, considering variable Relative Biological Effectiveness (RBE) via MONAS.

**Results:**

The findings show that helium and carbon minibeams offer enhanced protection of the normal tissues only ∼ 1–2 mm close to the tumor borders, while protons achieve an overall better sparing in the rest of the phantom for the 1E mode, when looking purely at the cell survival.

**Conclusions:**

Although protons achieved the highest mean cell survival in normal tissue, the actual sparing effect is strongly influenced by beam size and valley dose, with helium and carbon ions showing enhanced confinement of damage near the tumor edge. These findings highlight the need for the implementation of more anatomically accurate phantoms with more concise biological data as a basis.

## 1 INTRODUCTION

It was estimated that in 2020, there were approximately 10 million deaths due to cancer and 19.3 million new cancer cases, which are estimated to increase by 47%, reaching 28.4 million cases by 2040.^1^ About half of all cancer patients are likely to go through at least one course of radiotherapy, subsequently making radiotherapy an essential part of cancer treatment.^2^ In external beam radiotherapy, radiation is administered outside of the body and it represents the predominant modality of radiotherapy.^3^ Due to its well‐established effectiveness, broad availability, and cost‐effectiveness, treatment using X‐rays continues to be the most utilized radiotherapeutic method.^4^ To evaluate the risk of damage to healthy tissues, as well as to predict the efficacy of treatment in eliminating tumors, the Normal Tissue Complication Probability (NTCP) and the Tumor Control Probability (TCP) are used, respectively.^5^ The therapeutic window is the difference between the TCP and the NTCP, with modern irradiation modalities seeking to widen it.^6^ This can be achieved by spatial fractionation, an approach through which parts of healthy tissue are selectively spared from exposure to irradiation.^7^ Firstly, it was introduced in 1909 as GRID therapy using orthovoltage X‐ray beams; later, it advanced to microbeam radiotherapy using synchrotron‐generated X‐rays in the 1990s, and it took the form of submillimeter (X‐ray) minibeam radiotherapy in the 2000s.^8^, ^9^ The aforementioned modalities became known as Spatially Fractionated Radiation Therapy (SFRT), characterized by alternating high‐dose and low‐dose regions.^7^ Because of the physical and biological properties of protons and heavier ions, particle therapy has exhibited considerable potential in cancer treatment. Since 1946, when R.R. Wilson first proposed using accelerated protons for cancer therapy, the application of particle therapy has consistently progressed.^10^ The beneficial properties of particle beams stem from the Bragg peak phenomenon, where most of the energy of charged particles is deposited at a certain depth in tissue, thus reducing the damage to adjacent normal tissues. The spread‐out Bragg peak (SOBP) technique, which modulates the energy of the particle beam to ensure the therapeutic dose is evenly distributed across the tumor, further enhances accurate energy deposition. Aside from protons, heavier ions, like helium and carbon, were also clinically used in radiotherapy, since they deposit more energy while they traverse cells.^11^, ^12^ Heavy ions show less angular spread and less range straggling than protons but have extended tails in the energy deposition behind the Bragg peak. A main argument for using heavy ions is that they deposit higher energy loss per unit mass, known as Linear Energy Transfer (LET), resulting in a higher Relative Biological Effectiveness (RBE) for cell killing up to an LET of ∼ 70–200 keVµm^−1^. At higher LETs, the overkill effect occurs, leading to an RBE reduction. Thus, ions like helium, carbon, or in some cases oxygen are used in clinics, avoiding much heavier ions.^13^

In heavy ion therapy planning, the dose in the target region is optimized using an RBE‐weighted dose, correcting the physical dose based on the RBE.^14^ To calculate it, biophysical models depending on the linear quadratic (LQ) model, like the microdosimetric kinetic model (MKM) or the local effect model (LEM), are used. While in proton therapy, a constant RBE of 1.1 is applied in most clinical cases, the variability of carbon ion RBE poses great challenges for therapy planning.^13^ Almost 410,000 patients had received particle radiotherapy worldwide by the end of 2023. 350,000 cases were treated with protons, around 56,000 with carbon beams, and nearly 3500 with helium ions, pions, and other particles.^15^ Helium beams offer a good compromise between the properties of protons and carbon, paving the way to further research and improvements in particle therapy.^16^ In recent years, there have been intense efforts to incorporate spatial fractionation into particle radiotherapy. Proton minibeam radiotherapy (pMBT) is an innovative treatment modality combining the properties of proton beams with their Bragg peak type dose deposition, along with the advanced tissue sparing of spatial fractionation.^17^, ^18^ Using a submillimeter‐sized beam, the target is irradiated in a grid or planar beam arrangement with a center‐to‐center (ctc) distance in the millimeter range, creating a pattern of dose maxima and minima at the entrance channel, which enhances sparing of healthy tissue. As the beams traverse the matter, they broaden due to small‐angle scattering, and with appropriate optimization of ctc, they can homogeneously cover the tumor.^19^, ^20^ Preclinical studies comparing proton minibeams with broad beams show reduced side effects to healthy tissue,^21^, ^22^, ^23^ while there is also evidence of better tumor control.^24^ However, the beam spread eliminates the advanced protection in deeper healthy tissues next to the tumor region. Helium and carbon minibeams can potentially improve normal tissue sparing since they undergo less small‐angle scattering.^25^ Whether the reduced small‐angle scattering can counterbalance higher cell killing due to the higher LET of heavy ions compared to protons and due to nuclear breakup reactions, that increase dose deposition beyond the Bragg peak has not been clarified yet. Potentially, a lower physical dose is necessary to accomplish the same effect in the tumor.

In a former study, Sammer et al. showed that higher mean cell survivals in normal tissue areas can be accomplished when only the lower dose limit of the ICRU criteria^26^ is fulfilled, leading to a heterogeneous dose distribution in the tumor.^27^ In a sequential simulation study from Sammer et al.,^28^ they used planar proton minibeams and compared different longitudinally heterogeneous irradiation schemes, that is, non‐uniform dose distribution along depth in the tumor, to the typical SOBP mode. The results showed that heterogeneous schemes had greater mean cell survival in normal tissues than SOBP, while having the same minimum prescribed dose in the tumor. Especially, one of these modes was the Single Energy Distal‐Edge Bragg peak (1E) from opposing directions, revealing a simplified application method of pMBT. The construction of a preclinical irradiation facility for helium and carbon beams would contribute to fully exploring their prospects. Considering that this ambitious endeavor requires high subsidies, it is vital first to estimate the possible advantageous tissue‐sparing effects of these ions in longitudinal heterogeneous irradiation. This in silico study is designed to bridge this gap by comparing the normal tissue‐sparing of the proton, helium, and carbon ions by applying 1E interlacing minibeams with longitudinal dose heterogeneity in the tumor. Simulations were performed in a water phantom using TOol for PArticle Simulation (TOPAS)^29^ utilizing the MicrOdosimetry‐based modeliNg for RBE ASsessment (MONAS) tool to calculate cell survival fractions.^30^

## 2 METHOD

### 2.1 Water phantom and simulation setup

In previous studies, Sammer et al. used a water phantom, considering a centered 50 mm‐thick tumor surrounded by 100 mm of water from both sides.^27^, ^28^ The same setup was implemented in this study to ensure consistency in the results. A water phantom (20 × 20 × 250 mm^3^) made of standard G4_WATER was positioned within a vacuum environment. Carbon, proton, and helium planar minibeams were applied, assuming that they are generated via magnetic focusing to avoid secondary particles from collimation. The initial energies for protons and helium ions were chosen based on PSTAR and ASTAR databases,^31^ respectively, so that the beam range coincides with the distal edge of the tumor. An additional literature review was performed to determine the appropriate energy for carbon ions. Preliminary simulations confirmed the correct range of the beams. The beam properties, such as beam size (σ), beam divergence (σ’), energy, energy spread, and ctc impinging on the surface of the phantom, are summarized in Table 1 for each particle type. The beam size was chosen following the simulation setup of previous studies.^20^, ^28^ Since there are no clinical minibeam facilities and the beam size is considerably lower compared to existing clinical beams,^32^, ^33^ a large beam divergence was simulated, taking into account that the emittance should remain constant. All the TOPAS (Version 3.9, GEANT4 Version 10.07.p03) simulations included the following physics lists: “g4em‐standard_opt4,” “g4h‐phy_QGSP_BIC_HP,” “g4decay,” “g4ion‐binarycascade,” “g4h‐elastic_HP,” “g4stopping,” and a default range cut (0.05 mm) for all particles.

**TABLE 1:** Beam properties at the entrance of the phantom for each particle type.

Particle type	σ [mm]	σ’ [mrad]	Energy [MeV/u]	Energy spread	ctc [mm]
Proton (H^+^)	0.2	10	145	1 ‰	14.4
Helium (He^2+^)	0.2	10	145	1 ‰	7.2
Carbon (C^6+^)	0.2	10	276	1 ‰	3.6

### 2.2 Calculating dose distributions using topas

Dose scoring has been done in one dimension (z axis) and two dimensions (x and z) with a built‐in scorer of TOPAS (DoseToWater) and a binning resolution of 0.1 mm in both dimensions. Each minibeam had 10^6^ primary particle histories. Statistical uncertainties of the simulated dose were estimated from the bin‐wise particle counts as a function of depth. Only bins within a lateral distance of 2σ from the beam axis were considered, as contributions at larger radii are dosimetrically negligible while exhibiting very large relative uncertainties. Under this criterion, the relative statistical uncertainty remained below 2% at the entrance region and below 7% in the tumor region. These uncertainties are on the same order as experimentally reported uncertainties for microdosimetric dose measurements (∼ 3%–7%)^34^ and are therefore considered acceptable for our simplified digital twin of the dosimetric and biological system. In an experimental setting, additional uncertainty arises from the biological component, comprising aleatoric variability (e.g., cell‐to‐cell differences, cell‐cycle effects) and epistemic uncertainty (e.g., model simplifications such as 2D culture geometry). Consequently, the dose simulation uncertainty is sufficiently small that the simulation model can be meaningfully compared with, and challenged by, biological models. In all regions where ion‐specific differences are analyzed, the observed effects exceed the estimated statistical uncertainty.

To create the interlaced pattern of the minibeams, the dose distributions were properly replicated and mirrored in Matlab (Version R2025a, Mathworks), considering a specific ctc for each particle type.^35^ In the case of protons, the ctc was 14.4 mm, similar to Sammer et al.^28^ Based on the approximate relative lateral spread of helium (∼½) and carbon (∼¼) compared to protons, the ctc were decreased to 7.2 mm and 3.6 mm, respectively. In the following analysis, only an area covering a full ctc width, the so‐called unit cell (UC), located centrally, was considered. By this means, field‐edge effects reported with collimation as in Charyyev et al.,^36^ can be avoided. The dose distribution within this UC was then normalized to the minimum prescribed dose in the tumor volume. For comparability across geometries, the dose was selected such that cell survival in each tumor did not exceed 10%. The minimum dose necessary to achieve this condition was delivered to the tumor, while no upper limit was imposed on the maximum dose, aiming for a heterogenous dose distribution.

### 2.3 Microdosimetric spectra scoring

Zhu et al.^37^ developed a microdosimetric extension in TOPAS to simulate the microdosimetric spectra, which are an important radiobiological tool used in the MKM. The extension offers a lineal energy scorer when microdosimeters are applied. For this simulation study, spherical tissue‐equivalent proportional counters (TEPCs) were used to measure the probability distribution of lineal energy (y), given by $y=\frac{\varepsilon }{\bar{l}}\;,$ with ϵ: deposited energy by one event in TEPCs sensitive volume (SV). In case of spherical TEPCs, the mean chord length ($\bar{l}$) is given by $\;\bar{l}=\frac{4}{3}\;\times \mathrm{tissue}-\mathrm{equivalent}\;\mathrm{radius}$. The tissue‐equivalent radius is the central parameter of the MKM, defining the volume where the energy is deposited, which represents the radiation‐induced damage. Then, the data for the frequency probability density distribution f(y) result from the binning of the lineal energy, giving the microdosimetric spectra.^37^ These spectra seem to agree with the experimental data.^37^, ^38^ Since lineal energy results from a single event also, mean values are usually registered. Dose‐mean lineal energy ($\bar{y_{D}}$) is a broadly used quantity, given by $\bar{y_{D}}=\int_{0}^{\infty }yd\left(y\right)dy$, with d(y): dose probability density of lineal energy.^37^ The scorer size should ideally be on the submicrometer scale to coincide with the dimensions of the subcellular domain in the MKM framework. Achieving adequate statistics at these scales would require a significant increase in the primary histories, leading to prohibitive computational time. Thus, the SV radius and the tissue‐equivalent radius were set to 1 mm. Although this choice reduces spatial resolution, it remains an improvement over commercial TEPCs, which have a length in the centimeter range. Moreover, for sufficiently large spherical volumes (on the order of several micrometers or greater), the mean lineal energy approaches the macroscopic LET as stochastic fluctuations in energy deposition become averaged.^39^, ^40^, ^41^ The scoring range of lineal energy was 0.001–10000 keVµm^−1^ and the scorers were placed at the following depths (z‐axis): ± 115 mm, ± 70 mm, ± 50 mm, ± 40 mm, ± 35 mm, ± 30 mm, ± 27 mm, ± 25 mm, ± 24 mm, ± 22 mm, ± 20 mm, ± 10, and 0 mm (= tumor center). More scores were close to the tumor edges, which coincided with the beams’ range, aiming to better capture the spectra at those critical depths.

**FIGURE 1:**
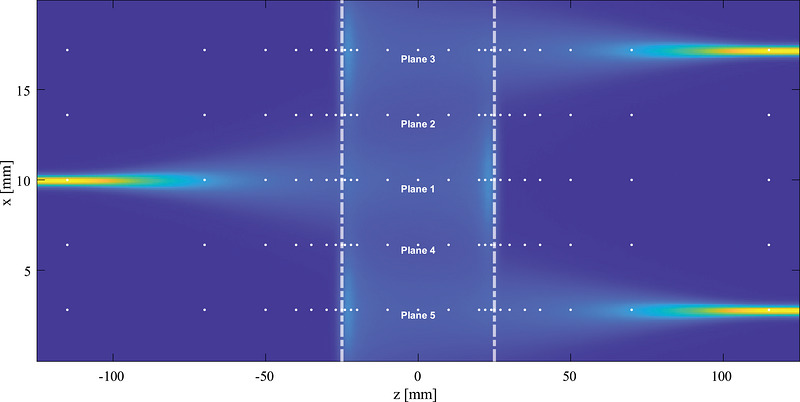
Longitudinal view of the phantom irradiated with three proton planar minibeams (parameters see Table 1) for microdosimetric spectra scoring. White circles show the locations of the scorers, situated at various depths across multiple lateral planes 1–5. The dashed white lines represent the tumor borders.

The simulation included three planar minibeams in the previously discussed interlaced arrangement to ensure that the scored spectra cover a full UC. The minibeams had the same parameters as in the case of the dose distribution simulations (see Table 1), as well as the same number of primary histories per beam. G4_WATER was selected to be the scorer's material to avoid wall effects, which occur in experiments with TEPCs. Regarding the lateral position of the scorers, five planes within the UC were chosen. One of them was in the middle of the minibeam (Plane 1), one at ctc/4 from each direction (Plane 2 and 4), and one at each direction of ctc/2 (Plane 3 and 5), which was the center of the opposed minibeam. The setup of microdosimeters within a phantom for proton irradiation is shown in Figure 1, while the same placement was done for helium and carbon. Scoring concentrated mostly on Planes 1 and 2 and Plane 3 was used to verify results in Plane 1, since the lack of adjacent minibeams in Plane 3 led to similar but distinct results to Plane 1. To avoid lengthy computational time, the mirrored version of Plane 1 was used for Planes 3 and 5, and the mirrored version of Plane 2 was used for Planes 4. The same seed was set for all the simulations, ensuring consistency in the outcomes.

### 2.4 Calculating cell survival using monas

MONAS is a toolkit of TOPAS developed to assess cell survival curves and dose‐dependent RBE, combining MC simulations of microdosimetric spectra with clinically applied radiobiological models.^30^ It is built on the lineal energy scorer of Zhu et al.^37^ and computes specific energy to apply it as an input in different models. The relation between lineal energy and specific energy (z), the desired quantity describing the dose in MKM, for a spherical region with radius r [µm] and density *ρ* = 1 gcm^−3^ is given by $z=\frac{0.16}{\rho \pi r^{2}}\;\cdot y$, where 0.16 converts keVg^−1^ to Gy^30^.

In this study, the saturation‐corrected MKM (MKM‐z*) was used. As noted in the previous section, the applied microdesimetry scorers were larger than the typical cell or nuclei. With this low spatial resolution, we intentionally chose a model that does not account for energy deposition in the cell domains or nuclei. MONAS provides the following default parameters for the human salivary gland (HSG) cell line, which were implemented in the study: *r*_d_ = 0.44 µm, *R*_n _= 3.9 µm, α_0_ = 0.19 Gy^−1^, β = 0.07 Gy^−2^, and y_0_ = 150 keVµm^−1^. Cell survival curves, which express the relationship between dose and cell survival fraction (SF), were created in MATLAB for each depth containing scorers by importing the MONAS output tables. These curves are described by the LQ model, SF(D) = exp(‐αD‐βD^2^). Thus, the linear and quadratic components, α and β, respectively, were determined for each depth. To calculate these components, and therefore the cell survival, for every voxel of the UC, a custom function was used. This function finds the nearest dosimeter sphere close to the voxel according to its coordinates and checks if the voxel's dose lies within ± 80% of the dose at the respective sphere. In this manner, the accuracy is increased, especially in areas with steep dose gradients, like next to or along a minibeam. If the criterion is not fulfilled, the function looks for the next closest sphere and repeats the same control. In case that dose is out of range, the function selects the α and β values of the original closest sphere. Detailed code for this process is provided in the supplementary file. At the end, the analysis results in maps of α and β values for each UC, and when combined with the dose distribution, the cell SF map is calculated for an entire UC.

## 3 RESULTS

### 3.1 Dose distributions

**FIGURE 2:**
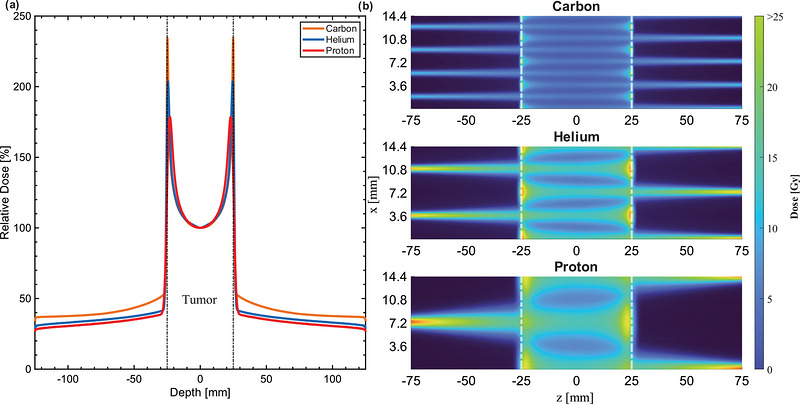
(a) Depth dose distribution for carbon (orange), helium (blue) and proton (red) beams. The dashed‐dotted lines show the tumor boundaries. Dose is normalized to the D_min_ within the tumor volume (b) 2D dose distribution for interlacing particle minibeams applied to achieve a maximum of 10% cell survival within the tumor. The tumor boundaries are indicated with the dashed‐dotted white lines. Dose values are color‐scaled up to 25 Gy. For better comparison, all of the distributions have a length equal to one proton UC.

Figure 2 shows the depth dose distribution for all three particles (cf. figure 2a), using data from the one—dimensional scoring, and the absolute dose distributions in two dimensions (2D) (cf. figure 2b). The dose is scaled to the minimum physical dose (D_min_) in the tumor for protons, helium and carbon ion beams. To achieve comparable results, the dose in each geometry was set to achieve a maximum 10% cell survival per tumor voxel, with a minimum dose in the tumor. These values vary from 2.4 Gy for carbon to 4.0 Gy and 4.4 Gy for helium and proton, respectively. Also, the mean dose in the tumor varies between the ions, from 3.7 Gy for carbon to 7.5 Gy for protons.

### 3.2 Microdosimetric spectra

**FIGURE 3:**
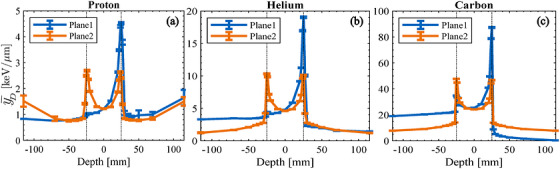
Dose‐mean lineal energy as a function of depth for interlacing minibeams: (a) protons, (b) helium ions, and (c) carbon ions. Dashed‐dotted lines indicate tumor boundaries.

In the next step, the microdosimetric spectra were scored in five planes of each UC. Figure 3 presents the dose‐mean lineal energy in depth for protons (a), helium (b), and carbon (c) minibeams. Due to the simulation design, Planes 3 and 5 show the same spectra as plane 1, flipped by 180°, whereas plane 4 has the same spectrum as plane 2. Therefore, only planes 1 and 2 are displayed in Figure 3.

The dose‐mean lineal energy of protons shows significant differences compared to those of helium and carbon. Overall, the dose‐mean lineal energy is much lower and reaches a maximum of 4.52 keVµm^−1^. Furthermore, in plane 1, the dose‐mean lineal energy increases at the rear end of the phantom compared to the entrance and directly behind the tumor. In plane 2, which is located between the interlacing beams, the dose‐mean lineal energy increases in both directions away from the tumor after having a minimum directly in front of and behind the tumor. In contrast, the spectra of the heavier ions show a decrease in dose‐mean lineal energy in all cases with greater distance to the tumor. For both helium and carbon, the dose‐mean lineal energy at the entrance is higher in the center of the beam (plane 1) than between the beams (plane 2). At the end of the beam's range in plane 1, the dose‐mean lineal energy is the highest with 19.04 keVµm^−1^ for helium and 87.37 keVµm^−1^ for carbon. Apart from an overall higher value for carbon than for helium, a striking difference is observed in the dose‐mean lineal energy distal to the tumor between planes 1 and 2. For helium, the spectrum is very similar, whereas for carbon, the dose‐mean lineal energy in plane 2 is considerably larger behind the tumor than for plane 1.

### 3.3 Cell survival

**FIGURE 4:**
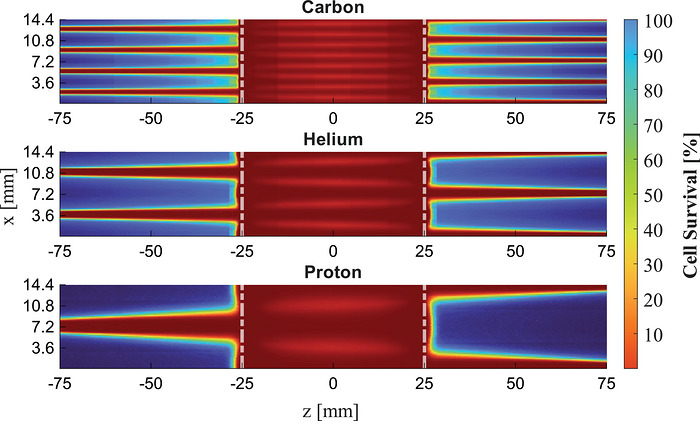
Cell SF maps for interlacing carbon, helium and proton minibeams. The D_min_ in the tumor is set to achieve a maximum of 10% cell survival within the entire tumor. White dashed‐dotted lines delineate tumor boundaries. For better comparison, all of the maps have a length equal to one proton UC.

**FIGURE 5:**
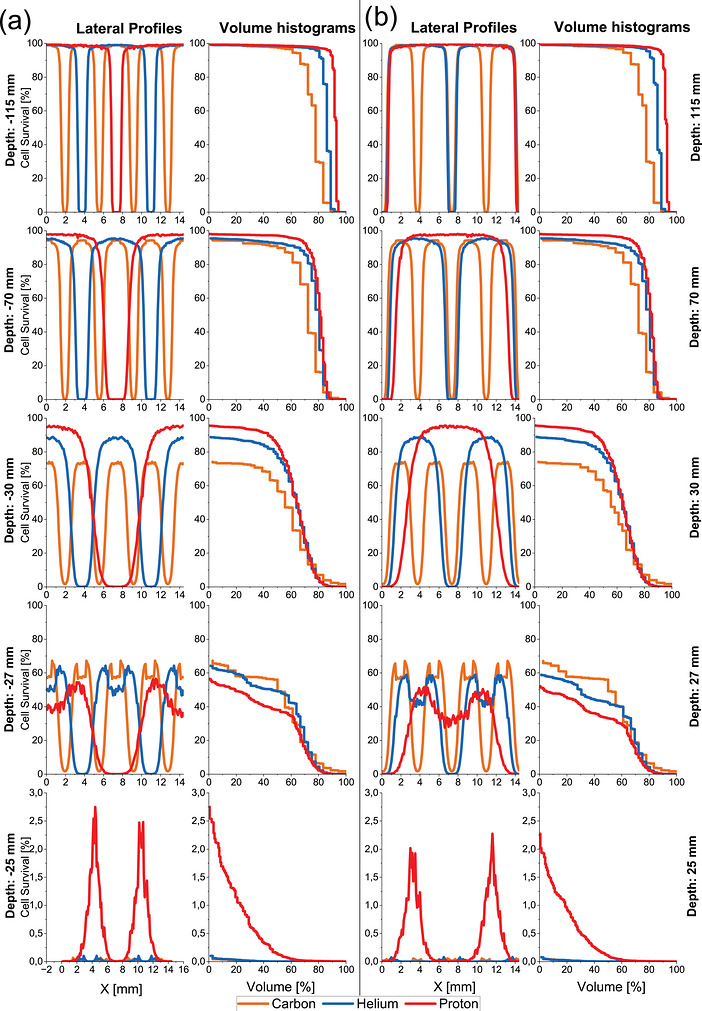
Lateral cell survival profiles and the cell survival volume histograms for specific depths within a proton UC for carbon (orange), helium (blue) and proton (red). The depths are ± 115 mm, ± 70 mm, ± 30 mm, ± 27 mm, and ± 25 mm (a) Profiles from the left side of the tumor (depths from −115 mm to −25 mm) (b) Profiles from the right side of the tumor (depths from +25 mm to +115 mm). The depths at ± 25 mm are the tumor borders where the cell survival is below 10%. Therefore, the *y*‐axis limit changed for visualization purposes.

The microdosimetric spectra were fed into the MONAS tool. This creates a cell survival curve for each of the placed microdosimeters, which can be fitted with the LQ model to obtain α and β values. Each scorer's corresponding cell survival curve is provided for planes 1 and 2 for all three ion species in the supplementary material. The survival curves in the Bragg peak region in plane 1 (24 and 25 mm) show a steeper gradient in all cases, compared to other depths. This effect is most pronounced in carbon and least in proton minibeam geometry. Overall, the steepness of the survival curves can be well correlated with the lineal energy and therefore LET present. The determined α and β values were used for each voxel to calculate the cell SFs from the 2D dose distributions, as shown in Figure 4. Additionally, for better comparison and evaluation, especially for regions close to the tumor edge, lateral cell survival profiles and cell survival volume histograms were calculated across each depth in one proton UC for all particle types. An app was designed with Matlab App Designer to generate these plots across the whole phantom. The app offers the opportunity to dynamically change or select the depth of interest and create the respective plot and will be provided in the supplementary material. In Figure 5, the lateral views and the volume histograms of cell survival are shown only for the following depths that contained scorers: ± 115 mm, ± 70 mm, ± 30 mm, ± 27 mm, and ± 25 mm.

**FIGURE 6:**
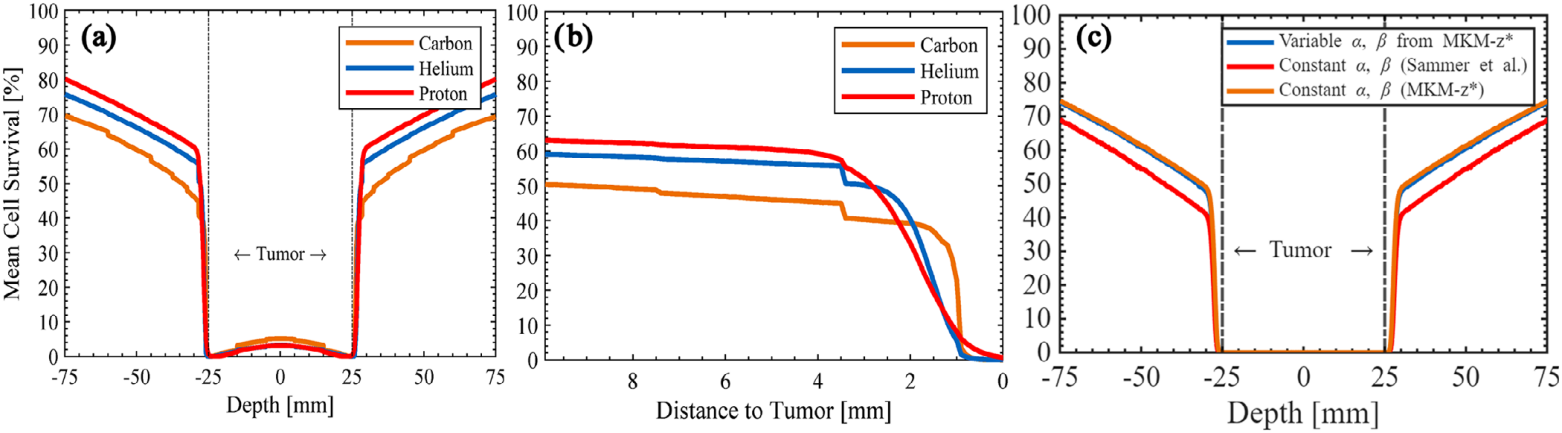
Mean cell survival as a function of depth within a UC for interlacing carbon (orange), helium (blue), and proton (red) minibeams (a) from 50 mm proximal to 50 mm distal to the tumor (b) 10 mm proximal to the tumor (c) Mean cell survivals for interlacing proton minibeams in the 1E mode with D_min_ = 9.75 Gy in the tumor and variable α and β values calculated by the MKM‐z* model (blue), are compared to two cases with the same D_min_ and constant α,β. Those from Sammer et al.(red)^28^ and the mean α,β of this study for the HSG cell line.

The results in Figure 5 indicate that close to the phantom entrance (± 115 mm), all particle types can achieve the same maximum cell survival. However, the volume histograms show that protons achieve this level for a larger region along the z‐axis, with helium and carbon following. In deeper depths, protons tend to preserve higher maximum cell survival for larger areas. Approaching the tumor edge, this behavior starts to change. At 2 mm next to the tumor from each side, carbon shows higher cell survival with helium and protons following. Additionally, the rectangular shape of the lateral cell survival profiles of carbon and helium beams at the depths of ± 27 mm indicates the effects of fragmentation. At the tumor borders at ± 25 mm depth, carbon and helium show almost no cell survival, while protons preserve a low percentage of cell survival up to ∼2.5%–2.7%. Both borders show similar cell survival profiles independent of beam direction, and they differ only by particle type. Furthermore, from the 2D cell SF maps, the mean cell survival per depth was calculated by averaging over each column of voxels from 50 mm proximal to 50 mm distal to the tumor (Figure 6a).

Also, these findings indicate that proton minibeams maintain the highest mean cell SF in normal tissue up to ∼3 mm from the tumor boundary. In comparison, helium and carbon ions provide additional normal tissue‐sparing effects closer to the tumor edges, extending approximately 1 and 2 mm, respectively (cf. figure 6b). Carbon ion minibeams result in relatively lower mean cell survival in normal tissues close to the tumor (< 1 mm), yet exhibit the highest mean cell survival in the middle of the tumor. This suggests a relatively higher dose heterogeneity in the tumor, simultaneously with a potentially smaller room to further increase the ctc while maintaining a maximum of 10% cell survival across all tumor regions. Nevertheless, their higher radiobiological effectiveness enables a reduced mean dose in the tumor to achieve the same effect. Helium ions offer a balance between the higher effectiveness of carbon ions and the superior tissue‐sparing properties seen with protons. Finally, the mean cell SF of protons with variable α,β is compared to two cases of constant α,β. The first set was the parameters from the study by Sammer et al^20^, ^28^ and the second was the mean α,β of the values calculated in this study for the HSG line. All of the cases are scaled to a minimum tumor dose of 9.75 Gy, like the scenario of Sammer et al.^28^ Figure 6c compares the mean cell SF across depths ranging from 50 mm proximal to 50 mm distal to the tumor for proton minibeams. The results indicate that the datasets are consistent only in the depth range of the tumor, where cell survival is very low. A noticeable difference of ∼6% in cell survival of normal tissues until ∼4 mm before the distal dose falloff region near the tumor is observed between the curve of constant parameters by Sammer et al,^28^ and the curves of variable and constant parameters calculated in this study. This discrepancy can be attributed to the differing methodologies used: Sammer et al.^28^ applied fixed radiobiological parameters (α = 0.425 Gy^−1^, β = 0.048 Gy^−2^) uniformly across the entire UC based on averaged data from all human cell lines within the Particle Irradiation Data Ensemble (PIDE).^42^ In contrast, the current study employed variable parameters (α = 0.202 ‐ 0.285 Gy^−1^, β = 0.070 Gy^−2^) derived specifically for the HSG cell line using the MKM‐z* model. Averaging these α,β parameters of this study (α = 0.2142 Gy^−1^, β = 0.07 Gy^−2^) leads to results similar to those with the variable parameters since they are for the same cell line, β is the same and α has only a small variation for protons. In the supplementary material, we present a plot comparing the mean cell SF with that of Sammer et al.^28^ α,β parameters with minimum tumor dose scaled to 9.75 Gy with the original data from Sammer et al.^28^ Implementing the MKM‐z* model allows for an analysis that considers differences in energy deposition at different locations. The ability to account for regional variations likely provides a more nuanced representation of biological responses. The observed discrepancy highlights the challenges in obtaining accurate model parameters for estimating radiobiological responses.

## 4 DISCUSSION

This study aimed to evaluate cell survival rates applying interlaced proton, helium and carbon minibeams in the 1E mode. This single energy interlaced mode was selected due to its simple delivery, as it is already highlighted by Sammer et al.^28^ It is the first time that minibeam effects for ions were simulated not only by scoring the dose but also by trying to take the biological effects coming from the complex energy spectrum, especially in the distal edge of the Bragg peak, into account. This was done using the MONAS tool, which can simulate cell survival curves for complex simulated micodosimetric spectra. Generally, helium, and carbon ions have less lateral spread compared to protons, resulting in smaller beam sizes along the beam path. It was assumed that this effect, in combination with the lower dose required to reach the same 10% cell survival within the tumor, may compensate for the increased potential of healthy tissue impairment due to higher LET. The results on mean cell survival indicate that these ions do not offer a significantly improved healthy tissue‐sparing with heterogeneous dose distribution in the 1E mode compared to protons. The cell survival volume histograms highlighted that proton minibeams seem to preserve an overall higher cell survival for larger areas, with helium and carbon following. Additionally, due to the interlaced irradiation scheme, the effects of fragmentation for helium and carbon minibeams tend to lower the cell survival rate next to the tumor borders. An advantage in healthy tissue protection using helium and carbon minibeams was only observed approximately 1 and 2 mm close to the tumor, respectively, with protons overcoming this ∼1 mm adjacent to the tumor edge. However, the heterogeneous dose distribution in healthy tissue might trigger other effects, which go beyond direct radiation‐induced effects. It is known from the research in the field of spatial fractionation with X‐rays that the beam size can have a big influence on the cell survival.^43^ A similar effect was shown in a proton beam study.^23^ Although the underlying mechanisms are not yet clear, this effect might be attributed to damage to the vasculature^44^ or dose‐volume effect^45^ on a small scale. Furthermore, it seems that the valley dose plays a key role in the reaction of tissue, where already low doses trigger severe damage in the whole tissue.^46^ This adds another level of complexity to the whole picture of particle minibeam effects. Taking these experiments into account, there might be a potential additional sparing of healthy tissue expected from the helium and carbon ion minibeams used in that study.

While this was the first study to take advantage of more complex modelling of the biological effects, the real data clearly show the limitations that such studies still face, due to a lack of proper data and models. Firstly, the results presented apply to the simple phantom model which consisted of a uniform water medium, which is a common setup.^47^, ^48^ Although this configuration yields valuable insights, it does not include the lateral heterogeneities found in real tissues, nor does it account for the complex boundary effects. A realistic clinical case considers layers of materials with different densities and compositions, such as air, bone, and muscle, leading to particle scattering. Therefore, the resulting dose distribution deviations observed in real tissues are not represented in a homogeneous water phantom. Minibeams with high‐LET particles such as helium and carbon ions, which are more affected by variations in tissue composition along their trajectories, may be more sensitive to heterogeneities. Consequently, even though the presented simulation results offer a fundamental understanding, they may change in more complex setups involving heterogeneous tissues and different tumor geometries. Furthermore, the ctc distance of the presented results is, in the case of protons, chosen based on previous studies,^28^ while for helium and carbon ions, the ctc was scaled down based on the lateral spread difference compared to protons. This approach was adopted because the primary objective of this first simulation study was to establish a simulation methodology implementing advanced radiobiological models that account for variations in particle energy deposition. Ideally, a comprehensive process would include a ctc optimization process based on the acceptable dose heterogeneity in the tumor. It should also be noted that ideally, the microdosimetric quantities are scored in micrometer—sized volumes. However, in this study, aiming to maintain manageable computational times, the radius of the SV and the tissue—equivalent radius are both set to 1 mm. A more severe limitation lies in the available models for radiation damage, which lack biological data. Eventually, effects for heavy ions are extrapolated from x‐ray data, even though the physical dose distribution is different and it's known that the mechanisms and effects are different.^49^ Furthermore, the model used in this study only provides data for the HSG cell line, applying its variable radiobiological parameters throughout the phantom. The comparison of a model using variable radiobiological parameters with one applying constant parameters without considering a specific tissue highlights the importance of implementing correct biological models. A more precise model should incorporate different α and β parameters for the normal tissue and the tumor to better replicate a real case. This can be easily implemented in the current setup, given that the required data is available. Cartechini et al.^30^ determined the HSG cell line parameters by refitting the biological parameters to better reproduce the in vitro cell survival curves of the PIDE database. This recalibration was conducted because of differences between TOPAS and previous studies regarding the radiation energy deposition in the sensitive volume.^50^, ^51^, ^52^ A similar approach can be followed to derive the parameters for other cell lines while using the MONAS tool. Additional experimental data across diverse cell lines are essential to validate and support the simulation results. As discussed before, the radiobiological reaction is even more complex due to biological factors such as cellular repair, migration, and intraminibeam immune interactions, which currently are not implemented in any simulation toolbox.

## 5 CONCLUSION

Summarizing, this in‐silico study conducted a comparative analysis to evaluate the protection of normal tissue close to the tumor edge using proton, helium and carbon minibeams in the 1E mode while preserving a maximum 10% cell survival within the tumor. The results indicate that protons offer the best cell survival in normal tissues except for a few mm next to the tumor edge, where helium and carbon minibeams seem to be more beneficial. Nevertheless, these findings are for an oversimplified phantom and the main objective of this study is to establish a simulation methodology. Future research efforts should focus on optimizing it and use a phantom with clinically relevant characteristics and a simulation model, which takes more comprehensive radiobiological models into account, since small beam sizes from helium and carbon, which are preserved deep in tissue, promise sparing. The study, therefore, highlights the importance of radiobiological models that account for variations in particle energy deposition across multiple levels of biological organization, as biological effects at the microscopic scale cannot be captured by the LQ model alone.

## AUTHOR CONTRIBUTION

**Aikaterini Rousseti**: performed data analysis, prepared figures, discussed the results, and wrote the manuscript. **Judith Reindl**: designed the study, prepared figures, discussed the results, and wrote of the manuscript. **Günther Dollinger**: designed the study, discussed the results, and reviewed the manuscript.

## FUNDING INFORMATION

This project was funded through BmVG project: präklinische Minibeam Anlage. We acknowledge financial support by Universität der Bundeswehr München.

## CONFLICT OF INTEREST STATEMENT

The authors have no relevant conflicts of interest to disclose.

## DECLARATION OF GENERATIVE AI

The authors used ChatGPT (OpenAI) for language refinement, text structuring, and preliminary information gathering on selected topics. Generated suggestions were critically assessed, verified, and partially adapted by the authors. After using this tool, the authors reviewed and edited the content as needed and took full responsibility for the content of the published article.
